# MyWSL: Malaysian words sign language dataset

**DOI:** 10.1016/j.dib.2023.109338

**Published:** 2023-06-22

**Authors:** Rina Tasia Johari, Rizauddin Ramli, Zuliani Zulkoffli, Nizaroyani Saibani

**Affiliations:** a Faculty of Engineering and Build Environment, Universiti Kebangsaan Malaysia, Selangor, Malaysia; b Faculty of Engineering, Technology and Built Environment, UCSI University, Kuala Lumpur, Malaysia

**Keywords:** Dataset, Hand gestures, Sign language, Image data

## Abstract

Deaf and hard-of-hearing individuals use sign language as a means of communication. However, those around them, especially family members like the children of deaf adults, may face communication challenges if they are unfamiliar with sign language. This issue has prompted numerous researchers to conduct studies on sign language translation and recognition. However, there is currently no publicly available dataset specifically for Malaysian sign language. This article introduces an image dataset of the Malaysian Sign Language (MySL) hand gestures used in everyday situations. The dataset, named MyWSL2023, comprises 3,500 images of ten static Malaysian sign language words collected from five participants (two males and three females) aged between 20 and 21 years old. The data collection took place indoors under normal lighting conditions. The MyWSL2023 dataset, which has been made freely accessible to all researchers, serves as a valuable resource for not only investigating and developing automated systems for hearing-impaired and deaf individuals but also gesture and sign language recognition using vision-based methods. The dataset can be accessed for free at https://data.mendeley.com/datasets/zvk55p7ktd.

**Specification Table**

**Table:** 

Subject	Computer Vision and Pattern Recognition
Specific subject area	Sign Language Recognition for the deaf and hard of hearing
Type of data	Image
How the data were acquired	The process of collecting images for this dataset involved instructing participants to sit in a relaxed manner within 1 meter of the camera and perform hand gestures. To capture the images, a 2.1-megapixel webcam equipped with a lighting control feature was utilized. This enabled precise and controlled image capture, ensuring the dataset would contain high-quality visuals that accurately depict the hand gestures.
Data format	The data organization consists of two sets of data. The first set comprises photographs in their original format (raw), while the second set consists of images from which unwanted background items have been.
Description of data collection	Images representing ten hand gestures for Malaysian Sign Language words - namely ‘water’, ‘fever’, ‘hear’, ‘eat’, ‘drink’, ‘wrong’, ‘I’, ‘quiet’, ‘sleep’, and ‘time’ - were included in the dataset. For each word, a total of 350 pictures were taken, resulting in a dataset comprising a total of 3,500 images.
Data source location	Faculty of Engineering and Built Environment, Universiti Kebangsaan Malaysia Bangi, Selangor Malaysia 43600 2.9289° N, 101.7800° E
Data accessibility	The dataset can be accessed at https://data.mendeley.com/datasets/zvk55p7ktd[1], and it is freely available to the public for research, academic, or educational purposes. Doi: 10.17632/zvk55p7ktd.1

## Value of the Data

• The availability of this data will assist researchers to develop more innovative techniques to enhance the Malaysian Sign Language recognition system.
• Advancements in this field greatly benefit society as they provide deaf individuals with a means to communicate using basic gestures and interact with the community.
• The proposed dataset could be utilized to develop a practical, user-friendly, and interactive application that translates Malaysian Sign Language. This application would have the potential to bridge the communication gaps between the deaf community and broader society, resulting in a more inclusive environment.
• The MyWSL2023 dataset serves as a foundation for the research community, providing a starting point for further exploration and development. It serves as a valuable resource upon which researchers could build because the dataset can be enhanced by incorporating additional image variations and diversities.

## 1 Objective

The dataset was generated to support researchers in their development of innovative techniques to enhance the automatic recognition of MySL gestures. Advancements in this domain have substantial societal advantages as they enable effective communication for the deaf community, allowing them to convey daily-life messages to people around them. This dataset can serve as a benchmark for fundamental MySL hand gestures, facilitating comparative evaluations and performance assessments. It can be expanded in the future as it can incorporate updated samples of gestures captured from various perspectives and environments. Such enhancements would enable researchers to make further progress and refine sign language recognition techniques.

## 2 Data Description

Online datasets for Malaysian Sign Language content are hard to obtain [2]. In this work, we developed the Malaysian Word Sign Language (MyWSL) dataset with the aim being to recognize 10 sign words that are commonly used in daily life. The dataset comprises RGB images of hand gestures corresponding to the following Malaysian Sign Language words: ‘water’, ‘fever’, ‘hear’, ‘eat’, ‘drink’, ‘wrong’, ‘I’, ‘silent’, ‘sleep’, and ‘time’. These are frequently used for everyday communication. Each word in the dataset represents a static hand gesture. Example datasets can be found in Refs. [3,4].

Although skin color variations may not be significant, the data collection process was conducted meticulously to include individuals with a wide range of skin tones. This was done to examine how well humans can recognize gestures made by those with different skin tones. In some instances, the skin tone may resemble background coloring, including the clothing worn by individuals, which could have a major impact on the classification accuracy. Therefore, all the images in this dataset were captured indoors, specifically in a controlled lab environment with various lighting conditions. Achieving high rates of gesture recognition is crucial as these gestures are vital for communication with deaf individuals in various contexts.

The study involved five Universiti Kebangsaan Malaysia students proficient in Malaysian sign language. Aged between 20 and 21, they comprised two males and three females who represented the diverse ethnic groups of Malaysia. The participants were recorded on camera performing various sign language tasks. Despite the small sample size, the study encompassed a substantial number of gestures, with each gesture class comprising 350 instances. Data collection occurred in a controlled laboratory environment and with a camera equipped with adjustable lighting settings. This enabled the capture of images under different lighting conditions, thus simulating various indoor lighting scenarios. By incorporating these lighting variations, the dataset became more diverse and representative of real-world conditions. Consequently, the trained model is expected to generalize well and perform effectively in various indoor lighting scenarios.

**Fig. 1:**
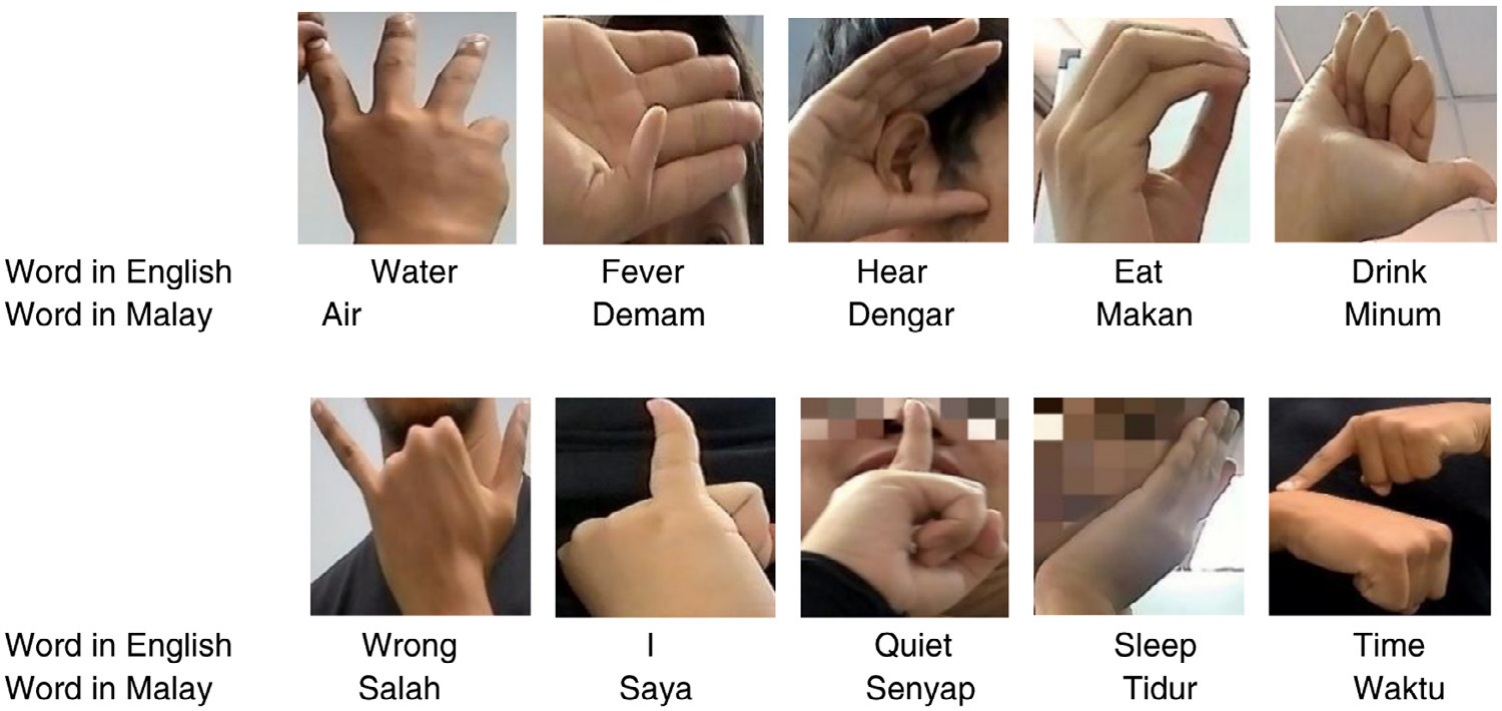
Gestures for the ten Malaysian Sign Language words.

**Fig. 2:**
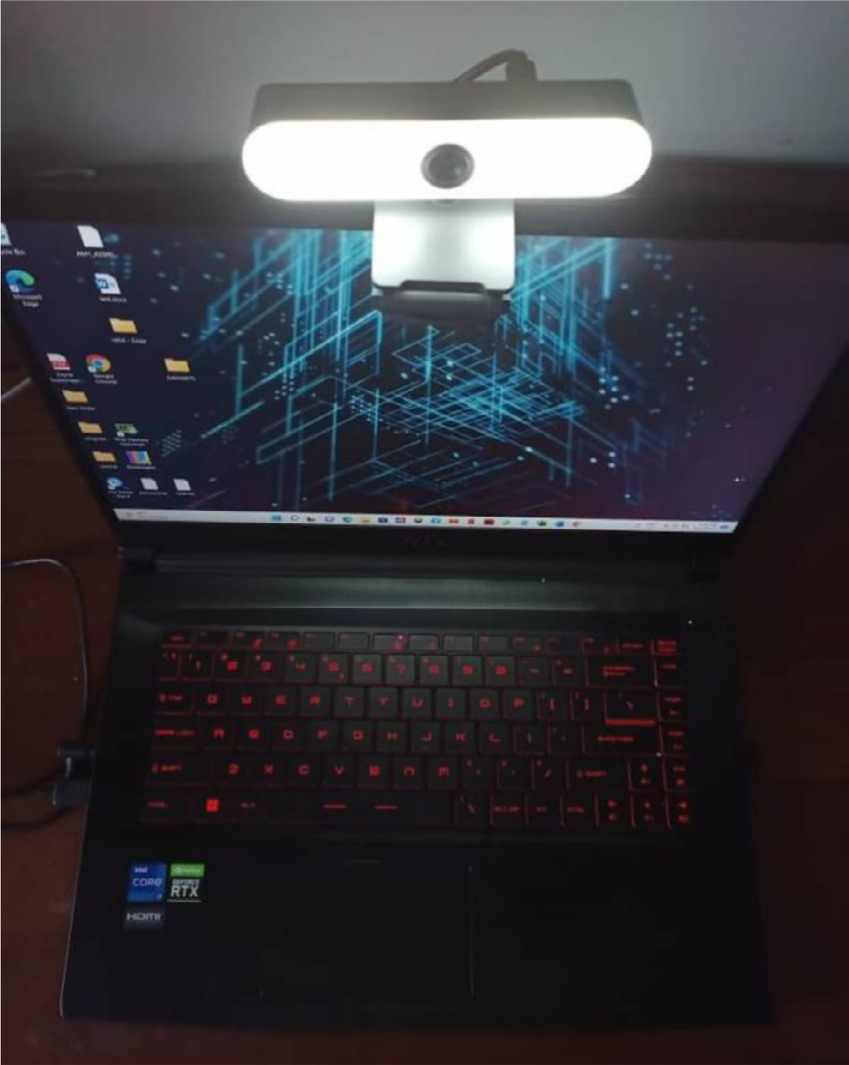
Web camera setup for the collection of sign language words.

**Fig. 3:**
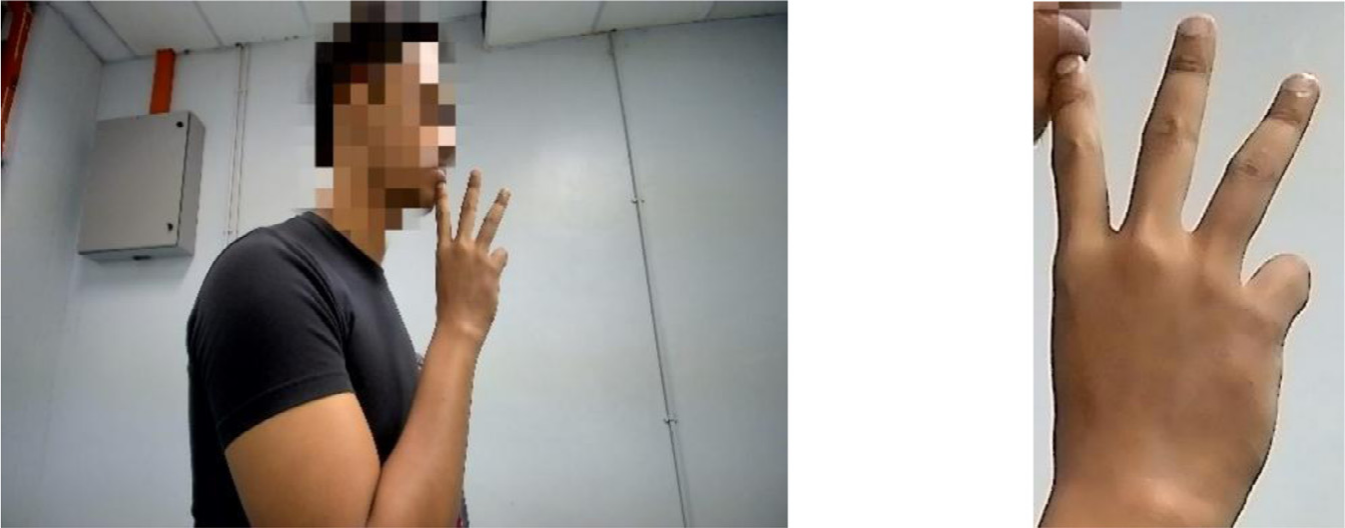
(a) Example of raw data of ‘water’ sign, (b) Example of crop data of ‘water’ sign.

The dataset is organized into two folders: ‘MyWSL2023 RAW DATA’ and ‘MyWSL2023 CROP DATA’. The former contains the original MyWSL images with dimensions of 1920 × 1080 pixels. The latter contains images that have been cropped to remove excessive background features. Sample images of all ten hand gestures from the ‘MyWSL2023 CROP DATA’ are shown in Fig. 1. Fig. 2 displays the laptop web camera setup used for collecting sign language words. Fig. 3(a) and Fig. 3(b) show examples of the word ‘water’ in its unprocessed (raw) state and subsequent cropped state, respectively. Table 1 presents the image classification performance using the simple Convolutional Neural Network method on the MyWSL cropped data.

## 3 Experimental Design, Materials, and Methods

The proposed system was made up of three phases:

(1) Dataset gathering
(2) Cropping images
(3) Data analysis

**Table 1:** Classification performance of simple CNN architecture on the MySL words in the cropped set.

MySL Word	Precision (%)	Recall (%)	F1-score (%)	Support (%)
Water	98	100	99	50
Fever	100	100	100	50
Hear	100	100	100	50
Eat	100	100	100	50
Drink	100	100	100	50
Wrong	91	100	95	50
I	100	100	100	50
Silent	100	88	94	50
Sleep	100	90	95	50
Time	91	100	95	50
				
Accuracy			98	500
Macro avg	98	98	98	500
Weighted avg	98	98	98	500

### 3.1 Dataset gathering

Images for the MyWSL dataset were collected from volunteers based at Universiti Kebangsaan Malaysia in an indoor environment. The pictures were captured using a web camera equipped with a lighting control feature, as shown in Fig. 2. The camera was positioned approximately one meter away from the volunteers. Multiple photos were taken, with the lighting conditions, perspectives, timings, and backgrounds varied to ensure diversity in the dataset. A total of 3,500 pictures were collected, with 350 images captured for each class. These pictures underwent pre-processing to prepare them for classification and recognition tasks.

### 3.2 Cropping images

The dataset was categorized into two directories: ‘MyWSL2023 RAW DATA’ and ‘MyWSL2023 CROP DATA’. The ‘MyWSL2023 RAW DATA’ directory contains unprocessed images with dimensions of 1920 × 1080 pixels. In the ‘MyWSL2023 CROP DATA’ folder, the images were processed to remove excessive background features, resulting in cropped versions. Fig. 3(a) displays a sample of the ‘water’ sign in its original form (raw), while Fig. 3(b) illustrates a sample of the ‘water’ sign after the cropping process.

### 3.3 Data analysis

The cropped dataset of MyWSL gestures was subjected to analysis through the implementation of a deep-learning approach using simple Sequential Convolutional Neural Network (CNN) architecture. For both training and testing, 86% of the dataset was used for training purposes, while the remaining 14% was allocated for testing.

The network underwent training for a total of 80 epochs. To evaluate the efficiency of the dataset, various performance metrics - such as precision, recall, F-score, and support - were employed for each gesture class. The results obtained from these evaluations are presented in Table 1. Table 1 highlights, the average precision, recall, and F1-score all achieved an impressive accuracy rate of 98%, indicating the model's strong performance.

## Ethics Statements

The collection contains hand gestures and does not include personal data. It was open-ended, and the volunteers used their hands willingly.

## CRediT authorship contribution statement

**Rina Tasia Johari:** Methodology, Validation, Writing – original draft. **Rizauddin Ramli:** Conceptualization, Resources, Investigation. **Zuliani Zulkoffli:** Writing – review & editing. **Nizaroyani Saibani:** Supervision.

## Declaration of Competing Interest

The authors declare that they have no known competing financial interests or personal relationships that could have appeared to influence the work reported in this paper.

## Data Availability

MyWSL2023 (Original data) (Mendeley Data).
